# *Angelica sinensis* Polysaccharides Ameliorate Stress-Induced Premature Senescence of Hematopoietic Cell via Protecting Bone Marrow Stromal Cells from Oxidative Injuries Caused by 5-Fluorouracil

**DOI:** 10.3390/ijms18112265

**Published:** 2017-10-28

**Authors:** Hanxianzhi Xiao, Lirong Xiong, Xiaoying Song, Pengwei Jin, Linbo Chen, Xiongbin Chen, Hui Yao, Yaping Wang, Lu Wang

**Affiliations:** 1 Laboratory of Stem Cells and Tissue Engineering, Chongqing Medical University, Chongqing 400016, China; xhxziris@outlook.com(H.X.); qrj1005@outlook.com(L.X.); zooey2213@gmail.com (X.S.); jingpw@outlook.com (P.J.); chenchen21310@gmail.com (L.C.); hjh931207@outlook.com (X.C.); wangkeke92@outlook.com (H.Y.); ypwangcq@aliyun.com (Y.W.); 2 Department of Histology and Embryology, Chongqing Medical University, Chongqing 400016, China

**Keywords:** 5-fluorouracil, *Angelica sinensis* polysaccharide, bone marrow stromal cell, hematopoietic cell, oxidative stress, aging

## Abstract

Myelosuppression is the most common complication of chemotherapy. Decline of self-renewal capacity and stress-induced premature senescence (SIPS) of hematopoietic stem cells (HSCs) induced by chemotherapeutic agents may be the cause of long-term myelosuppression after chemotherapy. Whether the mechanism of SIPS of hematopoietic cells relates to chemotherapeutic injury occurred in hematopoietic microenvironment (HM) is still not well elucidated. This study explored the protective effect of *Angelica sinensis* polysaccharide (ASP), an acetone extract polysaccharide found as the major effective ingredients of a traditional Chinese medicinal herb named Chinese Angelica (Dong Quai), on oxidative damage of homo sapiens bone marrow/stroma cell line (HS-5) caused by 5-fluorouracil (5-FU), and the effect of ASP relieving oxidative stress in HM on SIPS of hematopoietic cells. Tumor-suppressive doses of 5-FU inhibited the growth of HS-5 in a dose-dependent and time-dependent manner. 5-FU induced HS-5 apoptosis and also accumulated cellular hallmarks of senescence including cell cycle arrest and typical senescence-associated β-galactosidase positive staining. The intracellular reactive oxygen species (ROS) was increased in 5-FU treated HS-5 cells and coinstantaneous with attenuated antioxidant capacity marked by superoxide dismutase and glutathione peroxidase. Oxidative stress initiated DNA damage indicated by increased γH2AX and 8-OHdG. Oxidative damage of HS-5 cells resulted in declined hematopoietic stimulating factors including stem cell factor (SCF), stromal cell-derived factor (SDF), and granulocyte-macrophage colony-stimulating factor (GM-CSF), however, elevated inflammatory chemokines such as RANTES. In addition, gap junction channel protein expression and mediated intercellular communications were attenuated after 5-FU treatment. Significantly, co-culture on 5-FU treated HS-5 feeder layer resulted in less quantity of human umbilical cord blood-derived hematopoietic cells and CD34^+^ hematopoietic stem/progenitor cells (HSPCs), and SIPS of hematopoietic cells. However, it is noteworthy that ASP ameliorated SIPS of hematopoietic cells by the mechanism of protecting bone marrow stromal cells from chemotherapeutic injury via mitigating oxidative damage of stromal cells and improving their hematopoietic function. This study provides a new strategy to alleviate the complication of conventional cancer therapy using chemotherapeutic agents.

## 1. Introduction

Myelosuppression is a primary complication concern in patients undergoing chemotherapy. The hematopoietic system is organized in a hierarchical manner, in which the rare hematopoietic stem cells (HSCs) initiate the hierarchy and have the ability to self-renew, proliferate and differentiate into different lineages of peripheral blood cells through hematopoietic progenitor cells (HPCs) [1,2,3]. If hematopoietic progenitor cells (HPCs) are induced apoptosis and depleted by chemotherapy, acute myelosuppression occurs [4,5,6]. However, if HSCs undergo senescence with the ability of self-renewal impaired, a long-term damage to the hematopoietic system occurs [7,8]. The majority of chemotherapeutic agents can cause myelosuppression in a dose-dependent manner. Alkylating agents, pyrimidine analogs, anthracyclines, anthraquinones, nitrosoureas, methotrexate, hydroxyurea and mitomycin C are highly cytotoxic to bone marrow (BM) [8,9,10,11,12]. Following additional hematopoietic stress such as subsequent cycles of consolidation cancer treatment or autologous BM transplantation, long-term BM injury can deteriorate to become a myelodysplastic syndrome (MDS). Recent studies have reported that the MDS clone alters its local microenvironment suggesting a relationship between the BM microenvironment and HSCs depletion [13]. Moreover, a considerable susceptibility of human bone marrow stromal cells (hBMSCs) to chemotherapeutic drugs was demonstrated, and it was found that BMSCs cell death was induced at commonly used dose levels [14]. The role of BMSCs toxicity in drug-induced myelosuppression, rejection of stem cell transplants, and cell adhesion-mediated drug resistance suggests that in addition to HSC itself, the BM microenvironment may be impaired by chemotherapeutic agents, and this may be another reason for hematopoietic dysfunction [7,15,16,17,18]. Recent literature reported that in vitro expansion of hBMSCs combined with HSCs transfusion is an effective method of bone marrow hematopoietic reconstitution [19,20,21,22,23], however, the mechanism of chemotherapy-induced bone marrow hematopoietic microenvironment (HM) injury and its effect on the function of hematopoietic cells still need to be evaluated. Therefore, exploring the possibility and the underlying mechanisms to alleviate toxicity of chemotherapy in HM might be pivotal for long-term myelosuppression, and it might lead to new strategies for the screening of chemotherapeutic preventive agents.

Cells undergo stress-induced premature senescence (SIPS) after extensive replication or exposure to a genotoxic or oncogenic stress [24,25,26]. Reactive oxygen species (ROS), such as superoxide anions and hydrogen peroxide, are byproducts of normal oxidative metabolism in eukaryotic cells and are involved in many physiological signaling processes. However, an uncontrolled elevation of intracellular ROS levels and therefore accumulation of ROS induced-somatic oxidative DNA damage is believed to contribute to cellular aging and the senescence process [27,28,29,30]. To maintain genomic integrity, DNA repair and DNA damage response (DDR) are employed in cellular responses to oxidative DNA damage [29,31,32]. The most severe damage of oxidative DNA damage is DNA double-strand breaks (DSB) which activate the two major DDR pathways ataxia-telangiectasia mutated (ATM)-Checkpoint kinase 2 (Chk2) and ATM and Rad3-related (ATR)-Checkpoint kinase 1 (Chk1) and thereby trigger a series of signaling events including P53 to induce cell-cycle arrest [29]. These cells may resume to cell-cycle progression once damage has been repaired, or cells that suffered irreparable DNA damage undergo apoptosis or permanent cell-cycle arrest [28].

Anti-cancer drugs often augment the formation of ROS and associate with bone marrow cytotoxicity, myelosuppression, and immunosuppression [33,34,35]. Endosteal niches and vascular niches are two main components of bone marrow HM, which maintain low ROS level and provide a milieu for stem cell pool in hematopoietic homeostasis and during stress situation. BMSCs, as a heterogeneous cell population located in niches, are a crucial component of HM [36,37,38,39]. In the complex structure of the BM, HSCs are regulated by direct contact with BMSCs, by a variety of hematopoietic regulatory factors including cytokines and chemokines, and by extracellular matrix BMSCs produce. ROS stands at the cross-point of the regulation networks exerted on the stem cells and their adjacent cells [40,41,42,43,44,45]. HSC functions can be affected by the intracellular level of ROS that is produced endogenously through cellular metabolism or directly after exposure to exogenous stress such as chemotherapy.

Connexin-43 (Cx43) is highly expressed by BMSCs, osteoblasts, endothelial cells, and MSCs, and is also expressed by HSCs. Cx43 is the basic structural and functional protein of gap junction intercellular communication (GJIC) between BMSCs and HSCs. Cx43 maintains low ROS levels in HSCs and might thereby preserve HSCs function. Increased Cx43 expression was associated with a delay in differentiation of blood cells, resulting in increased production of hematopoietic precursors, while decreased Cx43 expression elicited an accelerated differentiation of myeloid precursor cells, suggesting that connexin-mediated coupling in the stroma modulates the ratio between proliferation and differentiation of hematopoietic precursors, therefore it is postulated to be a self-renewal gene. [46,47,48,49,50,51,52,53]. Cx43-deficient HSCs are prone to senescence because of their inability to transfer ROS to the HM leading to accumulation of ROS within HSCs [54]. Moreover, Cx43 expression in osteoblasts and osteogenic progenitors OB/P compartments is required for progenitor-dependent radioprotection/chemotherapy protection and serial transplantation. HM Cx43 deficiency impaired the migration of hematopoietic progenitors through irradiated stroma or 5-FU treated stroma, therefore elicited BM cellularity and hematopoietic progenitor content and delayed hematopoietic recovery after myeloablation with irradiation or 5-FU chemotherapy [49].

HSCs ROS levels are also influenced by a wide range of cytokines secreted from stromal supporting cells in the BM, which promote stem cell self-renewal, proliferation, differentiation, and migration [45]. One of the major players in maintenance of primitive cells is the cytokine stem cell factor (SCF). SCF-c-Kit signaling is needed for stem cell maintenance by reducing ROS levels in undifferentiated cells to maintain their primitive phenotype [55]. Stromal cell-derived factor (SDF-1) also named C-X-C motif chemokine 12 (CXCL12). CXCL12 is a cytokine that is essential for stem cell quiescence. It is produced and secreted by many different stromal cell types, especially CXCL12-abundant reticular (CAR) cells near the endosteum or sinusoidal endothelial cells and promotes normal hematopoiesis [51,56,57,58].Cell surface, membrane-bound via heparin sulfate CXCL12 is essential for stem cell quiescence, retention, and self-renewal when presented by the BM stroma [56,57,59,60]. On its secretion and release to the peripheral blood, it induces active stem and progenitor cell migration and mobilization that elevated by ROS, JNK, MMP9, etc. [61,62]. Oxidative stress can regulate CXCL12 and its receptor CXCR4. High levels CXCL12/CXCR4 signaling reduce HSCs ROS intracellular levels and induce quiescence. 

Various pro-inflammatory cytokines are produced during injury [63,64]. It has been reported that the over-production of pro-inflammatory cytokines, such as interferon (IFN) and and tumor necrosis factor (TNF), is one element of the activation of the oxidative DNA damage checkpoint mechanisms in HSCs [64,65,66]. Recent studies demonstrated that ex vivo treatment of HSCs with pro-inflammatory cytokine named regulated upon activation normal T cell expressed and secreted factor (RANTES) resulted in fewer T-cell progeny, and RANTES knockout mice rescued the aging-associated myeloid-biased lineage differentiation. RANTES is released from stromal cells in damaged tissue; binds to glycosaminoglycans on the endothelium; and attracts immune cells from the peripheral blood to sites of inflammation. RANTES induces expression of promyeloid transcription factors including Gata 2 which is important for the maintenance and expansion of multipotent progenitors and HSCs and reduces expression of lymphoid-specification genes including Ikaros and Gata3 which is shown to regulate early lymphopoiesis and T-cell development. Moreover, RANTES activates mTOR and its downstream target proteins such as P16, P19 and P21which lead to HSCs senescence [58,67]. Notably, those multiple factors mentioned above have innumerous pathways involved in hematopoiesis, stem cell mobilization, and immune system modulation, hence the regulation relationship among them may be complicated because of the pleiotropic effects in a specialized niche environment.

5-FU, an anti-metabolite with thymidylate synthase inhibitory activity, is commonly used in the treatment of high-proliferative, tissue-derived cancers, such as gastrointestinal carcinomas and aggressive breast cancer. Focused on oxidative stress, in this study human bone marrow-derived stromal cell line HS-5 was employed to investigate whether 5-FU may injure BMSCs and whether the injured BMSCs may degenerate hematopoietic function. The results showed that 5-FU significantly inhibited HS-5 growth. Both cellular apoptosis and senescence occurred in the stromal cells with the mechanism of oxidative damage induced by 5-FU. The expression of intercellular connexin protein was down-regulated in injured stromal cells coinstantaneous with declined function of intercellular communication. In addition, the levels of bioactive substances produced by stromal cells were altered with declined hematopoietic stimulating factors but enhanced inflammatory factors. Further, we established stromal cells and human umbilical cord blood-derived hematopoietic cells direct co-culture system, using HS-5 cells as feeder layer. Interestingly, the injured HS-5 feeder layer exposed to 5-FU impelled SIPS of hematopoietic cells. The present study preliminarily elucidated that the oxidative injured HM affected by chemotherapeutic agent may degenerate hematopoietic function.

ASP are major effective ingredients in Chinese Angelica with significant bioactivities including antioxidant, antitumor, hematopoietic regulatory, immunomodulatory, and radiation protective effects [68,69,70,71,72,73]. Our recent studies indicated extraordinary anti-aging roles of ASP which protected HSPCs against radiation-induced or d-Galactose-induced aging [74]. On this basis, our data clarified for the first time the protective effect of ASP on BMSCs from 5-FU injury, especially the protective effect on hematopoietic cells against SIPS via alleviating oxidative stress, preventing oxidative DNA damage, enhancing intercellular communication, and promoting hematopoietic stimulating factors originated from 5-FU injured BMSCs. 

## 2. Results

### 2.1. Tumor Suppressor 5-FU Had an Inhibitory Effect on BMSCs Growth

**Figure 1:**
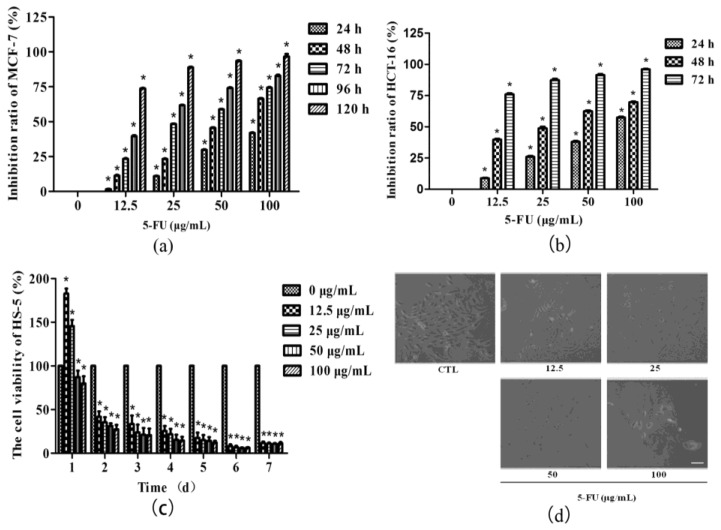
5-fluorouracil (5-FU) inhibits the growth of both tumor cells and bone marrow stromal cells in a dose and time-dependent manner. Cell proliferation assay was performed via the Cell Counting Kit-8 kit. (**a**) Cell inhibition rates are presented as (OD_control group_ − OD_experimental group_)/OD_control group_ × 100%. MCF-7 treated without 5-FU was used as a control; (**b**) The inhibitory effects of 5-FU on HCT-16 cells are presented as inhibition rates: (OD_control group_ − OD_experimental group_)/OD_control group_ × 100%. HCT-16 treated without 5-FU was used as a control; (**c**) The cell viabilities of HS-5 were almost entirely decreased with tumor-suppressive doses of 5-FU. Viability rate = OD_experimental group_/OD_control group_ ×100%. The cell viabilities of HS-5 without 5-FU treatment on each day were set as 100%. Results of HS-5 cell viability were normalized to the OD value of control HS-5; (**d**) Representative images of Fibroblast-colony forming unit (CFU-F) formed from 5-FU-treated and control HS-5 cells by phase contrast microscopy. CFU-F frequency decreased with increasing 5-FU doses (Scale bar = 50 μm). * *p* < 0.01 vs. control. CTL: Control; OD: optical density.

In this study, human breast adenocarcinoma cell line MCF-7 cell line and colorectal carcinoma cell line HCT-116 were selected to observe the suppressive effect of different concentrations of 5-FU in tumor cells. The results showed that from 12.5 to 100 μg/mL 5-FU significantly inhibited the growth of both tumor cells. The inhibitory effect was dose-dependent and time-dependent. Half of MCF-7 breast adenocarcinoma cells were inhibited growth after 25 μg/mL 5-FU treatment for 3–4 days, or 50 μg/mL 5-FU administration for 2–3 days (Figure 1a). In addition, 5-FU inhibited the growth of HCT-116 colorectal carcinoma cells distinctly. The inhibition rate was dropped to about 50% after 25 or 50 μg/mL 5-FU administration for 2 days (Figure 1b).

Human bone marrow stromal cell line HS-5 was then treated with 5-FU at its efficient tumor-suppressive doses to observe whether 5-FU may inhibit BMSCs growth. Cell Counting Kit-8 (CCK-8) results showed that there was no obvious inhibitory effect on HS-5 if exposed to a low dose of 5-FU and for a short-term, like 12.5 or 25 μg/mL 5-FU treatment for 24 h. However, increased dose of 5-FU and/or extended time for treatment could significantly inhibit HS-5 growth. The inhibitory effect was also dose-dependent and time-dependent (Figure 1c). Fibroblast-colony forming unit (CFU-F) represents a staminal compartment for the stromal precursors like mesenchymal stem cells (MSCs) and osteoblasts and sustains proliferation and differentiation of hematopoietic precursors. Damage or protection to this cellular compartment may affect the growth of BMSCs. Consistent with CCK-8 results, after in vitro culture for 12 days, the number of CFU-F exposed to different concentrations of 5-FU (12.5, 25, 50 and 100 μg/mL) were 122.67 ± 3.51, 80.33 ± 1.53, 42.67 ± 2.52 and 11.00 ± 1.00 respectively, significantly declined (*p* <0.01) compared with that of the control group (147.67 ± 6.03). The morphological changes of HS-5 with increasing 5-FU concentration were also apparently presented via microscope examination. The cell quantity in each colony was decreased concomitant with smaller and loosely dispersed shape (Figure 1d). The results suggested that 5-FU at tumor-suppressive doses exhibits a harmful role in BMSCs. Finally, 25 μg/mL 5-FU treatment on HS-5 for 48 h, as the minimum dose and minimum time for median inhibitory effect on tumors, was selected to continue the following experiments. 

### 2.2. Angelica Sinensis Polysaccharides Alleviated the Inhibitory Effect of 5-FU on BMSCs Growth

**Figure 2:**
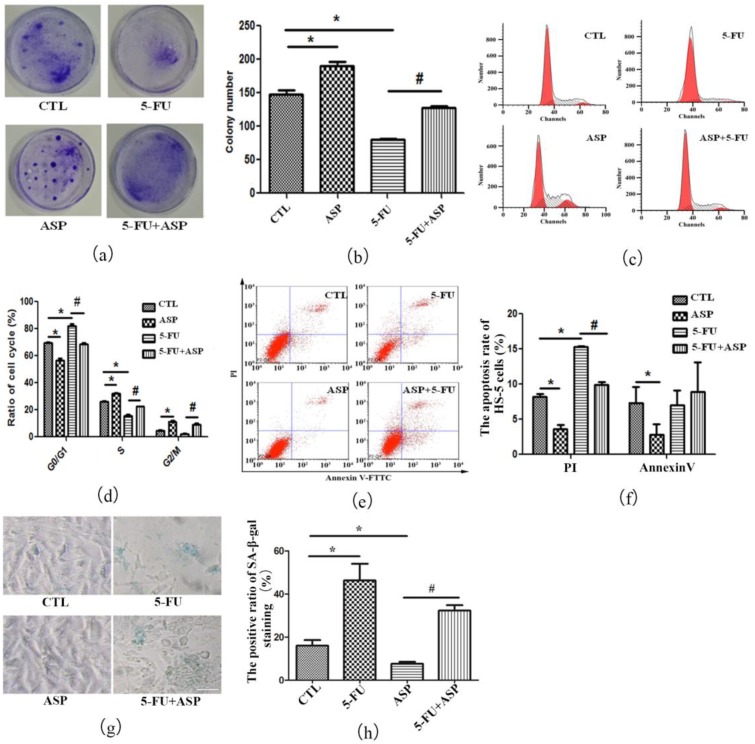
Angelica sinensis polysaccharide (ASP) rescues HS-5 cell growth inhibition after 5-FU treatment via anti-apoptosis and anti-senescence effects. (**a**) HS-5 cells treated with 5-FU, ASP, or a combination of both were cultured for 12 days, then stained by 0.5% crystal violet. CFU-F clusters are blue stained in dishes; (**b**) CFU-F frequency in different groups is presented as means ± SD; (**c**) Representative flow cytometry graphs of cell cycle analysis of HS-5 cells are shown; (**d**) The results of cell cycle distribution of HS-5 cells are presented as means ±SD; (**e**) Annexin V/Propidium iodide (PI) staining was employed to detect apoptotic HS-5 cells by flow cytometry. In each chart, upper left represents necrotic cells; bottom left represents vital cells; upper right represents intermediate-stage and late-stage apoptotic cells; and bottom right represents early-stage apoptotic cells; (**f**) The percentage of apoptotic HS-5 cells in different groups is presented as means ± SD. The left panel represents the ratio of intermediate-stage and late-stage apoptotic cells positively stained by PI. The right panel represents the ratio of early-stage apoptotic cells positively stained by Annexin V; (**g**) Senescence-related SA-β-gal staining was employed to detect senescent HS-5 cells. Senescent cells are blue-green stained (Scale bar = 50 μm); (**h**) The positive ratio of SA-β-gal staining is presented as means ± SD. * *p* < 0.01 vs. control group, # *p* < 0.01 vs. 5-FU group.

By crystal violets staining, each cell cluster representing CFU-F can be seen in a dish with naked eyes and the colony frequency can be counted. After administrated by 5-FU, the CFU-F frequency was significantly lower than that in control group (Figure 2a,b). Both cell death and cellular senescence are important responses to stress-associated stimuli. It was demonstrated that 5-FU exerts anticancer effects mainly by inhibiting tumor cell proliferation, activating Bax and P53, and/or caspase-dependent mechanism [74,75,76,77,78,79,80,81,82]. Annexin V/Propidium iodide (PI) staining assay is commonly applied to discern necrotic cells and apoptotic cells, further to quantify apoptosis. Loss of plasma membrane (PM) asymmetry is an early event in apoptosis, fluorescein isothiocyanate (FITC) labeled Annexin V interacts strongly and specifically with the exposed phosphatidylserine residues to quantify early-stage apoptotic cells. PI is impermeable to cells with an intact PM, hence until the cell integrity becomes compromised it gains access to the nucleus where it complexes with DNA rendering the nucleus highly fluorescent. The result of Annexin V/PI double staining chart was divided into four parts. Necrotic cells are Annexin V (−)/PI (+), whereas vital cells are Annexin V (−)/PI (−); early-stage apoptotic cells are Annexin V (+)/PI (−), whereas intermediate-stage and late-stage apoptotic cells are Annexin V (+)/PI (+) [82,83]. In our study, 5-FU caused HS-5 cell cycle arrest in G1 phase (Figure 2c,d) and a significant rise in apoptosis (Figure 2e,f), suggesting that apoptosis is a response for HS-5 stromal cells to 5-FU treatment the same as cancer cells respond to 5-FU. Moreover, although a very small number of documents suggest that senescence is also another cellular response to 5-FU [84], using senescence-specific SA-β-gal staining, a classical index for cellular senescence, we found an increase in positive ratio after 5-FU treatment (Figure 2g,h), suggesting that, apart from apoptosis, some survival HS-5 cells underwent senescence with permanent cell cycle arrest. Encouragingly, we found that ASP alleviated stress in HS-5 cell caused by 5-FU treatment. Compared with the control group, ASP treatment alone promoted HS-5 growth. Meanwhile, compared with the 5-FU group, the CFU-F frequency was resumed by ASP after 5-FU treatment. In addition, cell cycle arrest was ameliorated, and cellular apoptosis and senescence ratio were remarkably reduced. It was hinted that ASP may rescue human BMSCs growth inhibition after 5-FU chemotherapy via inhibiting apoptosis and ameliorating senescence.

### 2.3. Angelica Sinensis Polysaccharide Alleviated Oxidative Stress in BMSCs Caused by 5-FU

**Figure 3:**
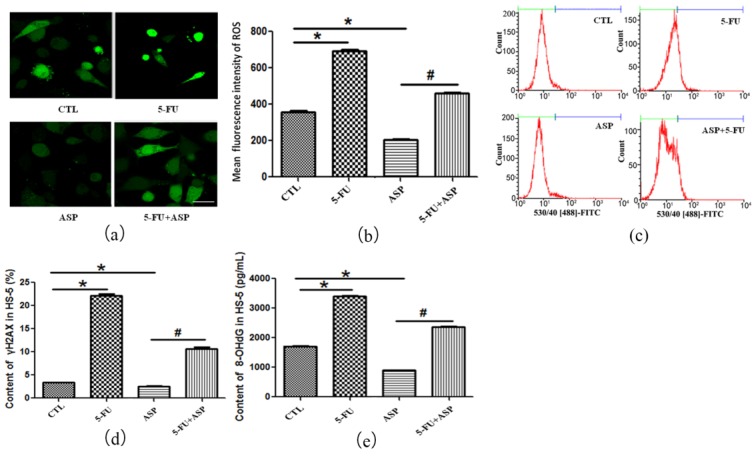
ASP alleviates oxidative stress caused by 5-FU in BMSCs. (**a**) The levels of reactive oxygen species (ROS) in HS-5 cells were measured by dichlorodihydrofluorescein diacetate (DCF-DA) staining and fluorescence microscopy (Scale bar = 50 μm); (**b**) The mean fluorescence intensity of ROS was quantified and is presented as means ± SD; (**c**) DSB of DNA was determined by γH2AX flow cytometry. Representative flow cytometric images of γH2AX in HS-5 cells are presented. The green line represents γH2AX negative cells and the purple line represents γH2AX positive cells; (**d**) The results of γH2AX content in HS-5 cells determined by flow cytometry are presented as means ± SD; (**e**) DNA damage response was detected by 8-OHdG supernatant Enzyme-linked immuno sorbent assay (ELISA). The results of 8-OHdG content in HS-5 cells are presented as means ± SD. * *p* < 0.01 vs. control group, # *p* < 0.01 vs. 5-FU group.

Under physiological conditions, the intracellular ROS production and scavenging of ROS via antioxidant enzymes are in dynamic equilibrium. Cells will be in oxidative stress state if ROS increase and/or the activity of antioxidant enzymes declines. The present study suggested that 5-FU weakened the antioxidant ability of HS-5 and put the cells under oxidative stress as data showed that after 5-FU treatment the content of ROS in HS-5 was remarkably higher than that of the control group (Figure 3a,b), however, the levels of antioxidant enzymes including GSH-Px and SOD were obviously decreased (Table 1). γH2AX is a recognized indicator of double-stranded DNA cleavage and γH2AX foci is commonly used as a DNA damage marker [85]. 8-OHdG is also a specific product of DNA damage response (DDR) [75,86,87]. In the 5-FU group, the levels of γH2AX and 8-OHdG in HS-5 cells were both significantly higher than those in the control group (Figure 3c–e), suggesting that oxidative stress induced by 5-FU could lead to DDR, which may cause BMSCs apoptosis or senescence.

In our study, ASP treatment alone exerted antioxidant effects on HS-5 compared with the control group, which is one of the classical properties of traditional Chinese medicine angelica. Meanwhile, after 5-FU treatment, ASP rebalanced the level of intracellular antioxidant enzymes and ROS, afterwards reduced γH2AX and 8-OHdG production. It was hinted that ASP may antagonize the oxidant burden 5-FU forced on the BMSCs via improving the cellular antioxidant activity and decreasing the high level of intracellular ROS and protect the cells from DNA damage. This may be the mechanism for how ASP rescues HS-5 cell growth inhibition after 5-FU treatment. 

### 2.4. Angelica Sinensis Polysaccharide Restored the Function of BMSCs after 5-FU Injury

**Figure 4:**
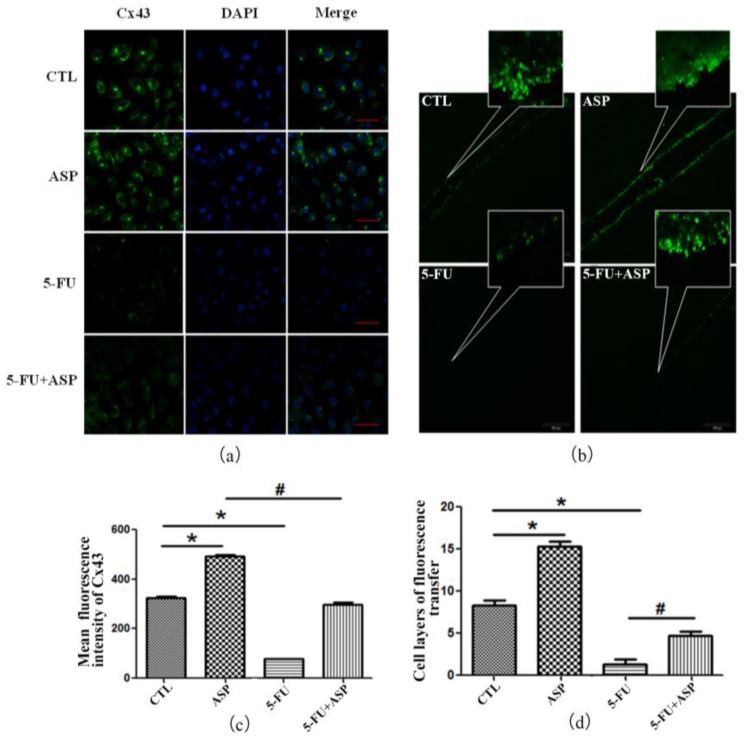
ASP reverses gap junction intercellular communication between bone marrow stromal cells after 5-FU injury. (**a**) Cx43 protein expression in HS-5 cells was detected by confocal laser scanning microscopy and is shown from a merged image of FITC-conjugated Cx43 and 4',6-diamidino-2-phenylindole (DAPI) staining of the nuclei in control (**top**), ASP-treated (**the second panel**), 5-FU-treated (**the third panel**) and 5-FU + ASP-treated (**bottom**) cells (Scale bar = 50 μm); (**b**) Scrape-loading and dye transfer assay was performed. The Lucifer Yellow transmission layers represent the capacity of intercellular communication; (**c**) Mean fluorescence intensity of Cx43 in HS-5 cells represented as means ± SD; (**d**) The cell layers of fluorescence transfer indicating the capacity of intercellular communication represented as means ± SD. * *p* < 0.01 vs. control group, # *p* < 0.01 vs. 5-FU group.

Gap junction (GJ) is a direct mode of communication between cells. Cx43 exerts a protective role and regulates the ROS content in HSPCs through ROS transfer to the HM, resulting in HSCs protection during stress hematopoietic regeneration. In addition, these GJ channels have the ability to transfer ions and low-molecular-weight secondary messengers to make BMSCs form a dynamic syncytium thus modulate the ratio between proliferation and differentiation of hematopoietic precursors. The most remarkable feature of Cx43 is their elevated expression in coincidence with the most active hematopoiesis at the sites of regeneration after cytotoxic treatment and in the epiphyseal marrow of growing bodies [46,49,50,88,89]. In our study, Cx43 protein expression and GJIC function of HS-5 were detected by immunofluorescence and SLDT assay. We found that both the expression of Cx43 (Figure 4a,c) and the transfer function (Figure 4b,d) in stromal cells were declined, demonstrating the injury process which 5-FU forced on the stromal cells. However, ASP regained Cx43 expression and restored the GJIC function of HS-5 dramatically.

**Figure 5:**
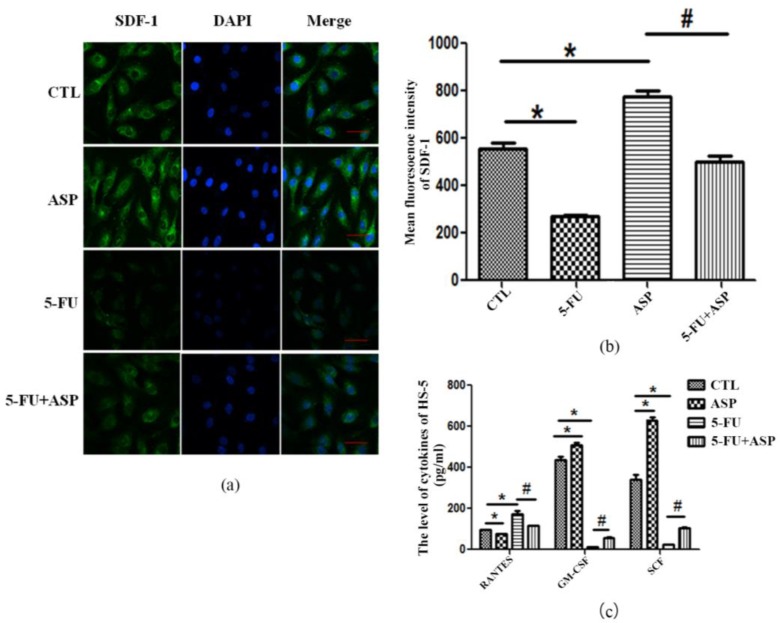
ASP recovers cytokines production from 5-FU injured bone marrow stromal cells: (**a**) SDF-1 (also named CXCL12) protein expression in HS-5 cells is detected via confocal laser scanning microscopy and shown from a merged image of FITC-conjugated SDF-1 and DAPI staining of the nuclei in control (**top**), ASP-treated (**the second panel**), 5-FU-treated (**the third panel**) and 5-FU + ASP-treated (**bottom**) cells (Scale bar = 50 μm); (**b**) Mean fluorescence intensity of SDF-1 was quantified and is presented as means ± SD; (**c**) (ELISA) assay was employed to detect the levels of cytokines produced by HS-5 cells. The results are presented as means ± SD. * *p* < 0.01 vs. control group, # *p* < 0.01 vs. 5-FU group.

Hematopoietic niches comprise supporting BMSCs that secrete an array of soluble and membrane-localized factors and create the unique microenvironment required for the maintenance and self-­renewal of HSPCs, as well as for their commitment and differentiation. Our data showed that 5-FU exposure led to attenuation of SDF, SCF, and GM-CSF produced and secreted by HS-5 cells, but elevated the level of RANTES. Meanwhile, after ASP treatment, the contents of SDF, SCF, and GM-CSF in HS-5 cells were significantly increased, however, RANTES secretion was decreased (Figure 5a–c). All the results mentioned above suggested that the stress in HM forced by 5-FU treatment may affect the function of BMSCs via inhibiting their intercellular communication and altering bioactive substances secretion. This may be the mechanisms that 5-FU retards hematopoiesis. However, ASP enhanced intercellular communication and promoted positive hematopoietic regulatory factors secretion by BMSCs. Hence, ASP may have a potential to restore hematopoietic function.

### 2.5. ASP-Treated HS-5 Feeder Layer Protected Co-Cultured Hematopoietic Cells from Oxidative Stress-Induced Premature Senescence

**Figure 6:**
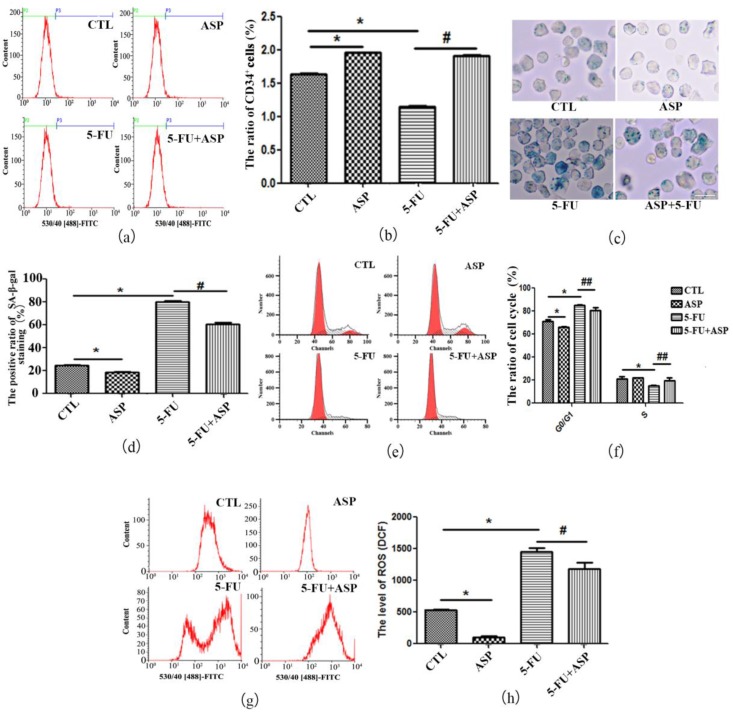
ASP-treated HS-5 feeder layer protects co-cultured hematopoietic cells from oxidative stress-induced premature senescence. (**a**) Survival CD34^+^ HSPCs co-cultured on HS-5 stromal cell feeder layers were examined via flow cytometry. The green line represents CD34⁻ cells and the purple line represents CD34^+^ cells; (**b**) The percentage of co-cultured CD34^+^ HSPCs in human umbilical cord blood-derived hematopoietic cells is presented as means ± SD; (**c**) The senescent hematopoietic cells were positively stained by SA-β-gal to be blue-green. Co-cultured on the 5-FU-treated feeder layer, the frequency of SA-β-gal positive hematopoietic cells increased. However, co-cultured on the ASP-treated feeder layer, the number of senescent hematopoietic cells reduced (Scale bar = 50 μm); (**d**) The percentage of SA-β-gal positive co-cultured hematopoietic cells is presented as means ± SD; (**e**) Representative flow cytometry graphs of cell cycle analysis of co-cultured hematopoietic cells; (**f**) Results of cell cycle distribution of co-cultured hematopoietic cells are presented as means ± SD; (**g**) Levels of ROS in co-cultured hematopoietic cells were measured by DCF-DA staining and flow cytometry; (**h**) Mean fluorescence of ROS was quantified and is presented as means ± SD. * *p* < 0.01 vs. control group, # *p* < 0.05 vs. 5-FU group, ## *p* < 0.01 vs. 5-FU group.

To verify the effect of injured BMSCs on hematopoietic function, we further co-cultured human umbilical cord blood-derived hematopoietic cells on conditional HS-5 stromal cell feeder layers. Counted by Trypan blue staining, the number of hematopoietic cells cultured on 5-FU administrated feeder layer was (0.96 ± 0.05) × 10^6^, which was significantly lower than that of the control group (1.75 ± 0.08) × 10^6^ (*p* < 0.01). Notably, among the hematopoietic cells, CD34^+^ HSPCs were also dramatically decreased (Figure 6a,b). Further detection demonstrated that co-cultured hematopoietic cells underwent senescence indicated as G1 phase arrest and positive SA-β-gal staining, decreased antioxidant enzymes and increased ROS content (Figure 6c–h and Table 2). As our expectations, the ASP treated stromal cell feeder layer promoted the growth of hematopoietic cells. The number of co-cultured hematopoietic cells was (2.05 ± 0.09) × 10^6^, which was significantly higher than the control group (*p* < 0.01), and among them CD34^+^ HSPCs maintained a relatively high level. ASP treated feeder layer reduced the intracellular ROS of hematopoietic cells and kept the hematopoietic cells in an active state. Moreover, compared with the 5-FU group, ASP remarkably alleviated the oxidative stress in hematopoietic cells co-cultured on 5-FU-administrated feeder layer, hence led to less senescent hematopoietic cells and the total number of hematopoietic cells rebounded to (1.27 ± 0.06) × 10^6^ (*p* < 0.01). Particularly the CD34^+^ HSPCs ratio was found to go up after co-culture. The results suggested that the 5-FU injured BMSCs have a significant suppressive effect on BM cellularity. The underlying mechanism may relate to oxidative stress-induced premature senescence of hematopoietic cells caused by the role of ROS in perturbation of stromal cell function upon aging, which in turn affects hematopoietic regeneration. ASP can ameliorate SIPS of hematopoietic cells with the possible mechanism that ASP may alleviate the exogenous oxidative stress in HM and then improve the function of stromal cells to relieve oxidative stress in hematopoietic cell.

## 3. Discussion

Bone marrow (BM) suppression is one of the most common complications of conventional cancer therapy using chemotherapeutic agents and irradiation. Exposure to chemoradiotherapy induces not only acute myelosuppression but also long-term BM suppression [90]. In contrast to acute myelosuppression, long-term BM suppression is manifested by a decrease in HSCs reserves and a defect in HSCs self-renewal; moreover, long-term BM suppression is long-lasting and exhibits little tendency for recovery. Previous studies have demonstrated that exposure to high doses of irradiation caused long-term bone marrow injury, in part, by selectively inducing HSCs senescence [91,92].

HSC aging has also become a concern in chemotherapy of older patients. HSC function declines with age, and prolonged myelosuppression in response to cytotoxic chemotherapy drugs suggests a reduced BM regenerative capacity in older individuals [93,94]. More recently, there is evidence to indicate a distinct role for intrinsic and extrinsic factors in HSC aging. Gene expression of HSCs is not a purely intrinsic process but is regulated by the interplay of different cell types of the stem cell niche and their functions. Computational modeling approaches, in combination with transplantation experiments, have established a critical link between niche aging and an indirect induction of HSC aging [21,95,96]. Mounting evidence elucidated that in addition to HSCs, the BM hematopoietic microenvironment has been reported to be impaired underlying the mechanism of adipogenesis after long-term chemotherapy [97,98]. However, the molecular mechanisms that regulate the HM control on HSCs aging are poorly understood.

The specific cellular microenvironment in which HSCs reside within the BM cavity, termed a niche is required for correct HSCs function under basal and stress conditions. BMSCs are the most important component of niches encompassing a generic group of mesenchymal-lineage stromal cells such as osteoblasts, n-cadherin-preosteoblastic cells, CXCL12 abundant reticular (CAR) cells, osteoblastic progenitors, and so-called mesenchymal stem cells (MSCs), which have been shown to play a signaling role in the control of the composition and function of HSC niches [36,37,38]. HS-5 originated from human bone marrow stromal cells is a classical hematopoietic supporting cell line that is used to study hematopoiesis in vitro. In the present study, HS-5 was employed to elucidate the extent chemotherapeutic drug may injure HM and its related mechanism. We found that the tumor-suppressive doses of 5-FU inhibited the proliferation of BMSCs causing their apoptosis and senescence.

Oxidative stress occurs when ROS production exceeds the capacity of antioxidant systems to control ROS levels. Studies implicated the role of ROS in perturbation of stromal cell function upon aging, which in turn affects BM’s reconstitution ability in aged mice. Treatment of the mice with an antioxidant curcumin was found to partially rescued stromal cells from oxidative stress-dependent cellular injury and this rejuvenation of stromal cells significantly improved hematopoietic reconstitution in 18-month-old mice compared to age control mice [99]. In our study, we found that 5-FU weakened the antioxidant capacity of HS-5 cells, and the intracellular ROS content increased significantly. HS-5 cells were sensitive to high level of ROS to undergo DDR which eventually caused HS-5 cells to undergo either apoptosis or senescence. Is the role of DDR, which arise from the stromal cells, one potential cause of hematopoietic suppression after chemotherapy? To elucidate this question, we established human umbilical cord-derived hematopoietic cells and HS-5 feeder layer co-culture system by placing normal donor hematopoietic cells in contact with stromal layers, and found that the HS-5 feeder layer injured by 5-FU treatment inhibited the growth of hematopoietic cells including CD34^+^ HSPCs with the underlying mechanism of SIPS. 

How does the oxidative stress in BMSCs initiate the oxidative stress in hematopoietic cells? The possible mechanisms were further studied. Documents have shown that Cx43 exerts a protective role and regulates the HSPCs ROS content through ROS transfer to the HM, resulting in HSCs protection during stress hematopoietic regeneration. That means during genotoxic stress a substantial fraction of newly generated ROS are eliminated through the Cx43 gap junction channels between HSCs and stromal cells, and they are deposited in niche cells rather than being confined to HSCs. Cx43 deficiency delays hematopoietic recovery after myeloablation with 5-FU. Without doubt, Cx43 is a major mediator of ROS scavenging through transfer from HSCs to stromal cells. However, since Cx43 is mainly expressed by BM stromal cells, osteoblasts, endothelial cells, and MSCs in niche microenvironment, Cx43 itself can be affected by oxidative burden from hematopoietic microenvironment. In vivo, antioxidant administration prevents the defective hematopoietic regeneration, as well as exogenous expression of Cx43 [99,100]. Thus, in our experiment, we further analyzed Cx43 expression and tested GJIC function in HS-5 cells under different circumstances. The expression level of Cx43 in 5-FU group displayed significantly lower than that in control group. Functional tests using SLDT assay showed that the function of GJIC in HS-5 cells treated with 5-FU was also dramatically impaired. These data above indicated that the oxidative burden exerted by 5-FU on stromal cells negatively regulated Cx43 activity which may be one cause of oxidative stress in hematopoietic cells. 

CXCL12 signaling can also limit ROS levels of HSCs. High levels of membrane-bound CXCL12 binding to CXCR4^+^ HSCs act to reduce HSCs ROS intracellular levels and promote ROS_low_ HSCs quiescence and retention; While low CXCL12 levels during G-CSF induced mobilization and stress due to enhanced proteolytic enzyme activity and degradation of osteoblasts promoted ROS_high_-enhanced HSC proliferation, myeloid differentiation, and migration potential. When CXCL12 was deleted, HSCs translocated from the BM endosteal area to areas around the sinuses demonstrated by function deletion experiment. The high perfusion area of the sinuses promoted ROS production in HSCs because of the elevated oxygen levels in the microenvironment. Moreover, loss of CXCL12 resulted in a reduction of the cytokine SCF, and its receptor c-Kit, whose signaling was also shown to reduce ROS levels in HSCs [60,101]. Similar to the effect of ROS on Cx43, ROS are involved in niche-mediated growth factor/chemokine receptor signaling through regulating its ligand expression. Low level of ROS promotes CXCL12 presentation on the MSC membrane. Significantly, CXCL12 activates several signaling pathways in stem cells, particularly the survival kinase, PI3K/Akt, which is also an important mediator of connexins. On the other hand, BMSCs form a dynamic syncytium via connexin gap junctions that regulates CXC12 secretion. Intercellular GJ channels in the BM stroma permit the transfer of small noncoding RNA, such as miR-197, a microRNA, which interferes with CXCL12 expression [102,103,104]. In our study, HS-5 cells expressed decreased CXCL12 after 5-FU treatment, this was simultaneous with the dropped Cx43 expression and function. Oxidative stress in HS-5 stromal cells caused by 5-FU impaired the ability of stromal cells to transfer ROS from HSPCs to HM, maybe this is one reason for SIPS of hematopoietic cells. Moreover, increased aging-associated inflammatory factors such as the pro-inflammatory RANTES also contributed to the hematopoietic SIPS. Notably, apart from the function of scavenging ROS from HSCs, intercellular gap junction like Cx43 and hematopoietic stimulating factors such as CXCL12, SCF, GM-CSF can also positively regulate HSPCs proliferation and differentiation by other signaling pathways. These factors were declined after chemotherapy and interplayed with each other as plausible mediators of dysfunctional interactions with hematopoietic cells; however, the precise roles of the network these factors fabricate are not well understood. 

Chinese angelica, a well-known traditional Chinese medicine, has been used to treat hematologic and gynecological conditions for centuries. It has been demonstrated that Angelica polysaccharide (ASP), an acetone extract polysaccharide found as the major active component in Chinese angelica, has various important biological activities, such as antioxidant, antitumor, hematopoietic regulatory, immunomodulatory, and radiation protective effects [105]. Literature indicated again the hematopoietic activity of ASP, which triggered human CD34^+^ HSPCs proliferation and differentiation under the mechanism that ASP stimulated spleen cells or peripheral blood mononuclear to secrete hematopoietic growth factors including IL-3, IL-6 and GM-CSF [106]. Moreover, the antioxidant properties of ASP protected the endothelial progenitor cells, hepatocytes, peritoneal macrophages and nerve cells from oxidative damage [107,108]. Our recent studies suggested that ASP protected mice against radiation-induced and d-Galactose-induced HSPCs senescence upon intrinsic factors of HSPCs including enhanced antioxidant ability and inhibited senescence-related signaling pathways. In the present study, extrinsic HM factors controlling regenerative hematopoiesis after chemotherapy have been investigated. Again, by the hematopoietic stimulating property and ROS scavenging property ASP exerted protective roles upon hematopoietic cell senescence. Interestingly, the mechanism of ASP alleviating SIPS of hematopoietic cells related to being niche-mediated via protecting HS-5 stromal cells from 5-FU-induced oxidative injuries. ASP reduced oxidative stress and oxidative DNA damage, boosted direct cell-cell contact between stromal cells and hematopoietic cells through Cx43 junctions, regulated cytokines, growth factors and chemokines such as CXCL12, SCF, GM-CSF, RANTES and thus provided a homeostatic microenvironment for HSPCs to regenerate the following myelosuppression. Generally, ASP as major constituents of initial extraction of the root, underlines the hematopoietic protective function of Chinese *Angelica Sinensis* to some extent, and it might lead to new strategies for the screening of chemoradiation therapeutic preventive agents.

## 4. Materials and Methods

### 4.1. Reagents

ASP (Purity ≥ 95%) was purchased from Ci Yuan Biotechnology Co. Ltd. (Xi’an, China) and dissolved in saline at the concentration of 20 g/L and sterilized by ultrafiltration. 5-FU was purchased from First Affiliated Hospital of Chongqing Medical University and dissolved in DMSO to make storage solution at the concentration of 12.5, 25, 50 and 100 mg/L. Fetal bovine serum (FBS) and Dulbecco’s Modified Eagle Medium (DMEM)/High Glucose were purchased from Gibco (Waltham, MA, USA). The SA-β-gal Staining and Reactive Oxygen Species Assay Kits were purchased from the Beyotime Institute of Biotechnology (Shanghai, China). The SOD and GSH-Px kits were obtained from Nanjing Jiancheng Bioengineering Institute (Nanjing, China). The antibodies against γ-H2AX were obtained from Cell Signaling Technology (Danvers, MA, USA). The antibodies against SDF-1 were purchased from Boster Biological Technology (Wuhan, China). The antibodies against Cx43 were purchased from Abcam (Cambridge, UK). The Human ELISA Kit for 8-OHdG, RANTES, and SCF were obtained from Neobioscience Biological Technology (Guangdong, China). Lucifer Yellow CH dilithium salt was purchased from Sigma-Aldrich (St Louis, CA, USA).

### 4.2. Cell Lines and Culture

Human bone marrow stromal cell line HS-5, human colorectal carcinoma cell line HCT-116 and human breast adenocarcinoma cell line MCF7 were purchased from American Type Culture Collection and cultured in DMEM medium containing 10% FBS, 2 mM l-glutamine and 100 mg/L of penicillin-streptomycin (Gibco, Waltham, MA, USA).

Human umbilical cord blood samples were collected into sterile bags containing citrate dextrose from healthy females who just underwent full-term delivery without clinical complications. All participants provided informed consent. This study was approved by the Ethics Review Committee of Chongqing Medical University. Mononuclear cells were separated on Ficoll-Hypaque density gradient as described previously.

Human umbilical cord blood-derived mononuclear cells (hUCBD-MNC) were co-cultured with HS-5 cell feeders according to the following procedures. Prepared HS-5 layers were divided into 4 groups (control, 5-FU-administration, ASP treatment, and 5-FU administration plus ASP treatment). Control group was cultured as routine; in the 5-FU group 0.025 g/L 5-FU administrated for 48 h; in the ASP group 0.1 g/L ASP treated for 48 h; in the 5-FU + ASP group, after 6 h pretreatment of 0.025 g/L 5-FU, 0.1 g/L ASP concomitantly treated for 42 h. In the co-culture system, hUCBD-MNCs were cultured in long-term culture medium in 37 °C, 5% CO_2_-humidified chamber. After 6 h, non-adherent hematopoietic cells were collected and plated directly on prepared HS-5 feeder layers in suspension in iscove’s modified dulbecco’s medium (IMDM) with 10% fetal calf serum (FCS) or long-term culture medium. After 48 h, the hematopoietic cells were used for subsequent experimental measurement. 

### 4.3. CCK-8 Cell Proliferation Assay

Cell proliferation assay was performed using the Cell Counting Kit-8. The cells were plated in 96-well plates at 2 × 10^4^ cells per well and cultured. At the indicated time points, the optical density (OD) at 450 nm was measured in triplicate wells using a microplate reader. The cell inhibition rates of MCF-7 and HCT-116 were calculated according to the formula: inhibition rate = (OD value of the control group—OD value of the experimental group)/OD value of the control group × 100%. The cell viability of HS-5 was also calculated according to the formula: viability rate = OD value of the experimental group/OD value of the control group × 100%.

### 4.4. Cell Cycle Analysis

In total, 1 × 10^6^ cells in each group were collected and fixed with 70% cold ethanol at 4 °C overnight. Then, the cells were washed with PBS and incubated with propidium iodide (PI) at 4 °C for 30 min in the dark and were subjected to flow cytometry to quantify the percentage of cells in each cell cycle phase.

### 4.5. Fibroblast Colony Culture and Count

The HS-5 cells were adjusted to the concentration of 5 × 10^4^/mL and seeded in 25 mm^2^ dishes at 37 °C, 5% CO_2_ incubator. When the medium was replaced on the seventh day, 5-FU and/or ASP were added and the culture stopped on the twelfth day. The Petri dishes were washed with sterile PBS for 3 times and fixed by 4% paraformaldehyde for 30 min then dyed by the prepared 0.5% crystal violet staining solution for 5–10 min. After microscopical examination, each cell cluster that contains more than 50 cells was considered as a colony [109]. The number of colonies was counted in 3 independent samples per dish each group. The dishes for crystal violet-stained cell clusters and the colony observed under inverted phase contrast microscope were photographed respectively.

### 4.6. Apoptosis Detection

Percentage of early and late apoptotic cells was analyzed by Annexin-V/PI assay. Cells were treated with 5-FU, ASP and a combination of both for 48 h. The cell concentration was adjusted (2–5) × 10^5^/mL. Five microliters of AnnexinV–FITC labeling and 195 μL cell suspension were mixed and placed at room temperature for 10 min. After rinsed 2 times, the cell blocks were added with 190 μL binding buffer and 10 μL 20 μg/mL PI solution. Finally, the cells were detected with flow cytometer (Bd, Franklin Lakes, NJ, USA). 

### 4.7. Senescence-Associated β-Galactosidase (SA-β-Gal) Staining

SA-β-gal staining kit was used according to the manufacturer’s instructions and our previously reported procedures. HS-5 cells were stained in situ. Hematopoietic cells were centrifuged, fixed, smeared onto coverslips and then stained. At minimum, 1000 cells were counted in 10 random fields to determine the percentage of SA-β-gal-positive cells. 

### 4.8. Detection of Oxidation-Associated Biological Indicators

The levels of intracellular ROS, SOD, and GSH-Px were detected as we have previously reported. ROS in HS-5 cells or hematopoietic cells of each treatment group were measured respectively by flow cytometry or confocal laser scanning microscopy. Briefly, Cells were loaded with 5 μM of 2′,7′-dichlorodihydrofluorescein diacetate (DCF-DA) and incubated at 37 °C for 30 min, and the intracellular concentration of ROS was determined by the intensity of DCF-DA staining. The peak excitation wavelength for oxidized DCF-DA was 488 and emission was 525 nm. HS-5 cells were collected, lysed and centrifuged to collect the supernatant. Then SOD activity and GSH-Px content were measured using the corresponding assay kits according to the manufacturer’s instruction.

### 4.9. Measurement of DNA Damage Markers

The levels of 8-OHdG in different groups were detected by an ELISA kit following the manufacturer’s instruction. The levels of γH2AX were determined by flow cytometric analysis. 

### 4.10. Determination of Cytokines

The supernatants of HS-5 cells were collected. SCF, GM-CSF as well as RANTES in each group were measured by ELISA according to the kit manufacturer’s instructions. For SDF detection, immunofluorescence was used. Cells were smeared onto coverslips by cytospin, then stained with anti-SDF-1 (1:500), a biotinylated goat anti-rabbit secondary antibody.

### 4.11. Analysis of Cellular Gap Junction Cx43

Cx43 protein expression and GJIC function in HS-5 cells originated from co-culture system were detected by immunofluorescence and SLDT (scrape-loading and dye transfer) assay. To test gap junction channel Cx43 function, HS-5 cells in each group were washed three times with PBS, and 1 mL 0.05% (0.05% *v*/*v* dissolved in PBS) Lucifer yellow was then scrape loaded with several scrapes using a 4.5# syringe. The dye solution was kept in the 37 °C chamber for 10 min and then discarded. The cell cultures were carefully rinsed three times with PBS. Lucifer Yellow (457 Da)can pass through intercellular gap junction Cx43, so an inverted fluorescence microscope was employed to record the transmission of the dye from the edge cells of the scrape. An average value of 9 measurements for each treatment (3 measurements per dish) was regarded as the transmission of dye in the cell cultures.

### 4.12. Statistical Analysis

Statistical analyses were carried out using SPSS 19.0 software. Data are presented as the mean ± SD. One-way ANOVA was used for comparison of mean values across the groups and multiple comparisons were made by LSD test. Differences were considered significant at *p* < 0.05. 

## 5. Conclusions

In summary, ASP relieve SIPS of hematopoietic cells by the mechanism of protecting bone marrow stromal cells from chemotherapeutic injury ,this may be related to the inhibition of oxidative damage of stromal cells and improving their hematopoietic function. This study provided an experimental basis for the SIPS of hematopoietic cells, which contributes to further study to alleviate the complication of conventional cancer therapy using chemotherapeutic agents.

## Figures and Tables

**Table 1:** The levels of ROS, T-SOD, and GSH-PX in HS-5 cells ($\bar{X}\pm S$, *n* = 3).

Group	ROS (Flow Cytometry)	T-SOD (U/Mgprot)	GSH-Px (U/Mgprot)
Control	599.33 ± 10.21	103.20 ± 1.00	59.61 ± 0.85
5-FU	947.33 ± 6.66 *	8.60 ± 1.11 *	5.61 ± 0.38 *
ASP	92.00 ± 6.25 *	152.27 ± 1.00 *	85.41 ± 1.27 *
ASP + 5-FU	825.33 ± 24.54 ^#^	108.50 ± 0.90 ^#^	34.24 ± 1.13 ^#^

* *p* < 0.01 vs. control group, # *p* < 0.01 vs. 5-FU group.

**Table 2:** Effect of ASP regulated injured HS-5 cells on oxidation resistance in human umbilical cord blood-derived hematopoietic cells($\bar{X}\pm S$, *n* = 3).

Group	T-SOD (U/Mgprot)	GSH-Px (U/Mgprot)
Control	237.24 ± 0.91	270.55 ± 0.91
ASP	283.86 ± 0.86 *	367.21 ± 0.95 *
5-FU	174.68 ± 0.85 *	6.45 ± 0.41 *
ASP + 5-FU	202.44 ± 0.98 ^#^	156.53 ± 0.98 ^#^

* *p* < 0.01 vs. control group, # *p* < 0.01 vs. 5-FU group.

## Abbreviations

5-FU	5-fluorouracil
SP	*Angelica sinensis* polysaccharides
BMSC	Bone marrow stromal cell
Cx43	Connexin 43
CXCL12	C-X-C motif chemokine 12
CFU-F	Fibroblast-colony forming unit
DCF-DA	Dichlorodihydrofluorescein diacetate
DDR	DNA damage response
D-Gal	D-galactose
DMEM	Dulbecco’s Modified Eagle Medium
IMDM	Iscove’s Modified Dulbecco’s Medium
DSB	DNA double-strand break
FBS	Fetal bovine serum
GM-CSF	Granulocyte-macrophage colony-stimulating factor
GJ	Gap Junction
GJIC	Gap junction of intercellular communication
GSH-Px	Glutathione peroxidase
HM	Hematopoietic microenvironment
HSC	Hematopoietic stem cell
HSPC	Hematopoietic stem/progenitor cell
hUCBD-MNC	Human umbilical cord blood-derived mononuclear cells
MSC	Mesenchymal stem cell
OD	Optical density
PI	Propidium iodide
RANTES	Regulated upon activation normal T cell expressed and secreted factor
ROS	Reactive oxygen species
SA-β-Gal	Senescence-associated β-galactosidase
SCF	Stem cell factor
SIPS	Stress-induced premature senescence
CTL	Control
